# Diagnostic and Therapeutic Implications of Long Non-Coding RNAs in Leukemia

**DOI:** 10.3390/life12111770

**Published:** 2022-11-02

**Authors:** Vladimir Gasic, Teodora Karan-Djurasevic, Djordje Pavlovic, Branka Zukic, Sonja Pavlovic, Natasa Tosic

**Affiliations:** Laboratory for Molecular Biomedicine, Institute of Molecular Genetics and Genetic Engineering, University of Belgrade, 11042 Belgrade, Serbia

**Keywords:** long non-coding RNA, acute myeloid leukemia, acute lymphoblastic leukemia, chronic myeloid leukemia, chronic lymphocytic leukemia

## Abstract

**Simple Summary:**

Research on leukemia has often led to novel approaches in clinical practice. Cytogenetical and molecular markers have been introduced in treatment protocols contributing to better stratification of leukemia patients in specific prognostic groups and patient-tailored therapy. However, the next step concerns modern hematology. Omics-profiling of leukemia patients is needed to complete the information of all key players which influence the course of the disease. There is no doubt that regulatory RNAs, among which lncRNAs, belong to those key players. Knowledge of the role of lncRNAs in leukemias is not sufficient and the research in that field needs to be increased. This would enable the design of innovative targeted-therapeutics, thus opening the doors wide for personalized medicine in hematological malignancies.

**Abstract:**

Leukemia is a heterogenous group of hematological malignancies categorized in four main types (acute myeloid leukemia (AML), acute lymphoblastic leukemia (ALL), chronic myeloid leukemia (CML) and chronic lymphocytic leukemia (CLL). Several cytogenetic and molecular markers have become a part of routine analysis for leukemia patients. These markers have been used in diagnosis, risk-stratification and targeted therapy application. Recent studies have indicated that numerous regulatory RNAs, such as long non-coding RNAs (lncRNAs), have a role in tumor initiation and progression. When it comes to leukemia, data for lncRNA involvement in its etiology, progression, diagnosis, treatment and prognosis is limited. The aim of this review is to summarize research data on lncRNAs in different types of leukemia, on their expression pattern, their role in leukemic transformation and disease progression. The usefulness of this information in the clinical setting, i.e., for diagnostic and prognostic purposes, will be emphasized. Finally, how particular lncRNAs could be used as potential targets for the application of targeted therapy will be considered.

## 1. Leukemia

**Figure 1:**
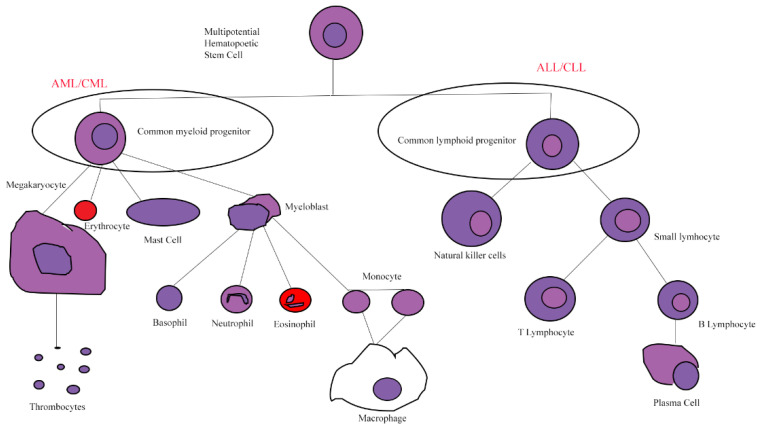
Rudimentary schematic of hematopoietic lineage. Leukemias are derived from the precursors lymphoblasts and myeloblasts.

Leukemias are malignant diseases of hematopoietic cells which result from abnormal proliferation and clonal expansion, aberrant differentiation and impaired apoptosis of the cell of origin, leading to the accumulation of leukemic cells in bone marrow and other hematopoietic tissues and suppression of normal blood cells’ production. Classification of leukemias is based on the predominant linage of the malignant cells, myeloid or lymphoid, while acute or chronic is based on the percentage of blasts or leukemic cells in bone marrow or blood (Figure 1).

### 1.1. Acute Myeloid Leukemia

Acute myeloid leukemia (AML) is the most common type of acute leukemia in adults and the second most common in children, accounting for approximately 80% and 20% of all reported acute leukemia, respectively [1,2]. Development of AML is characterized by unlimited proliferation and impaired differentiation of early myeloid cells leading to accumulation of immature blast cells in the bone marrow and peripheral blood, thus resulting in hematopoietic failure. The main feature of AML is its vast clinical and genetic heterogeneity. For example, recurrent chromosomal aberrations such as t(15;17) [PML-RARa], t(8;21) [AML1-ETO] and inv(16) [CBFB/MYH11] are frequent, but still around 50% of adult AML patients have a cytogenetically normal karyotype (AML-NK) [3,4,5,6]. Pretreatment karyotype analysis is the main feature used for risk-stratification in AML and based on this, patients are divided into favorable, intermediate and adverse risk groups [7]. Over the years, through advances in technology, other molecular markers have been identified and incorporated into the risk stratification and treatment strategy, such as mutations in fms-related tyrosine kinase-3 (FLT3), nucleo-phosmin (NPM1) CCAAT/enhancer binding protein (CEBPA) and isocitrate dehydrogenase 1 and 2 (IDH1 and IDH2) [8,9]. Changes in the expression pattern of certain genes were also recognized as significant in the diagnosis and therapy of AML [10,11,12,13]. However, it was only with the introduction of next generation sequencing (NGS) technology that a complete and comprehensive insight into the pathogenesis of AML was gained [14,15,16]. High-throughput transcriptome-wide profiling has enabled us to seek new biomarkers of AML beyond protein-coding genes and thus to focus on non-coding RNAs, specifically long non-coding RNAs (lncRNAs) and their roles in regulation of main cell processes such as differentiation, proliferation and cell cycle.

### 1.2. Acute Lymphoblastic Leukemia

Acute lymphoblastic leukemia (ALL) is the most common acute leukemia of childhood and the second most common acute leukemia of adulthood [17]. It is a malignancy of hematopoietic tissue characterized by increased proliferation of lymphocyte precursor cells, lymphoblasts, which accumulate in the bone marrow and the peripheral blood [18]. One of the primary milestones in risk stratification of ALL is the division into two groups of ALL: B-Cell precursor ALL and the T-Cell precursor ALL. The most common genetic features in ALL are recurrent translocations such as t(12;21) [ETV6-RUNX1], t(1;19) [TCF3-PBX1], t(9;22) [BCR-ABL1] and chromosomal rearrangements involving MLL gene [3]. Even though chromosomal aberrations are frequent in ALL, they do not represent a single event that causes proliferation of the malignant clone [19,20,21]. Indeed, other molecular alterations involved in domination of the malignant clone and the development of full-blown ALL were detected. Many of them have been found to be important not only in the pathogenesis of the disease, but also in risk stratification and therapy and are already used in day-to-day clinical practice of ALL [22].

Precise stratification into different risk groups based on specific clinical and genetic characteristics is a guide for ALL treatment. Although the cure rates for childhood ALL approaches 90%, survival in the adult ALL population is only about 40% and decreases with age [23,24]. Since lncRNAs have already been shown to influence the course of ALL [25], lncRNAs could be the next step in improving diagnosis and treatment of ALL in both adults and children.

### 1.3. Chronic Myeloid Leukemia

Chronic myeloid leukemia (CML) is a dominant type of myeloproliferative neoplasm, accounting for about 15% of newly diagnosed leukemia in adults [26]. The main genetic event causing CML is reciprocal translocation t (9;22)(q34;q11), or Philadelphia chromosome, causing formation of “breakpoint cluster region” (BCR)-“Abelson murine leukemia” (ABL) fusion transcript, which encodes the BCR-ABL oncoprotein with constitutively active tyrosine kinase activity [27]. The first line of treatment for CML is the use of specific tyrosine kinase inhibitors (TKIs) such as imatinib mesylate (IM) that binds to the ATP-binding site of BCR-ABL preventing its conformational switch to the constitutively active form of oncoprotein, and thus causes blockage in uncontrolled proliferation of CML cells [28]. Despite of the enormous success of imatinib treatment resulting in the increased 8-year OS in as many as 80–90% of CML patients, in the remaining percentage of patients long-term exposure to TKIs can induce the development of drug resistance [29]. Patients that are resistant to TKIs experience evolution of the disease, passing from the chronic to the accelerated phase and finally to the blast crisis phase with all of the characteristics of acute leukemia and very poor prognosis [30]. Different mechanisms of resistance to TKIs treatment have been reported such as kinase-domain mutations, BCR-ABL overexpression and altered expression of drug transporters, but also some mechanisms that are BCR-ABL-independent that involve alternative activation of many downstream signaling pathways (MAPK, JAK/STA, PI3K/AKT) [31]. Based on the fact that some lncRNAs have already been identified as contributors to imatinib resistance, current studies recognize lncRNAs as a target for overcoming drug resistance in CML.

### 1.4. Chronic Lymphocytic Leukemia

Chronic lymphocytic leukemia (CLL) is the most common type of leukemia in adults in Western countries, which affects mainly elderly individuals (median age at diagnosis of 72 years), with a higher incidence in males (1.7:1) [3]. It is a malignancy of mature, functionally incompetent, monoclonal B lymphocytes with the common immunophenotype sIgM^weak^ CD5^+^ CD19^+^ CD23^+^ CD20^weak^ [32]. The extreme clinical heterogeneity of CLL, from indolent to rapidly progressive, treatment-resistance disease, reflects the diversity of mechanisms involved in its pathobiology.

CLL cells proliferate primarily in lymph nodes and accumulate in blood, bone marrow and secondary lymphoid organs due to intrinsic impairment of apoptosis and microenvironment-mediated signals. Complex interactions with the extrinsic factors from tissue microenvironment, including antigens, which trigger tonic B-cell receptor (BCR) and Toll-like receptor (TLR) signaling, activate anti-apoptotic and proliferative pathways and are crucial for CLL pathogenesis and evolution [33]. The importance of antigenic drive on the BCR as the key player in CLL biology is evidenced by strong prognostic significance of somatic hyper-mutational status of immunoglobulin heavy variable genes (IGHV) and BCR “stereotypy” [34].

The highly complex genomic landscape of CLL contains more than 2000 recurrent genetic alterations, which include chromosomal aberrations (deletion 13q14, trisomy 12q, deletion 11q22-q23, deletion 17p13), mutations of protein-coding genes, epigenetic modifications and alterations in non-coding RNAs [35,36,37]. Out of over 40 recurrently mutated genes identified in CLL, only several are mutated in more than 5% of patients at diagnosis (*NOTCH1, SF3B1, TP53, ATM*) [3]. Most of the mutated driver genes cluster in a set of signaling pathways involved in cell cycle control and DNA damage response (*ATM*, *TP53*, *POT1*), chromatin modifications (*HIST1H1E*, *CHD2*, *ZMYM3*), RNA processing (*SF3B1*, *XPO1*), transcription (*EGR2*, *IRF4*, *BCOR*, *MED12*), as well as in pathways mediated by microenvironment, such as NOTCH (*NOTCH1*, *FBXW7*), TLR (*MYD88*), MAPK (*BRAF*, *KRAS*, *NRAS*, *MAP2K1*) and NF-κB (*BIRC3*, *TRAF3*, *NFKBIE*) [33].

Since genetic and epigenetic heterogeneity of CLL only partially correlates with its clinical heterogeneity, and over the past decade the attention shifted towards the investigation of non-coding RNAs (ncRNAs) in CLL. Deregulation of all classes of ncRNAs has been observed, which are involved in various cellular processes altered in CLL, such as proliferation, apoptosis, genomic instability, interactions with microenvironment and angiogenesis [38].

## 2. Long Noncoding RNAs

Long noncoding RNAs (lncRNAs) are products of transcription from a DNA template of length greater than 200 base pairs (bp) which do not translate into a polypeptide or functional protein. Despite not undergoing translation, lncRNAs participate in numerous processes, which influence cell function, genomic regulation, transcription and translation [39].

By development of high-throughput technologies it was determined that only 2% of the human genome accounts for coding genes and that the rest of the genome is comprised of so called “junk DNA”, or non-coding DNA. Newest data from RNA-seq analysis have shown that the large portion of non-coding RNAs (ncRNAs) accounts for lncRNAs. According to the comprehensive database of collection and annotation of noncoding RNAs (NONCODEv6) over 1,000,000 lncRNAs have been identified and around 18,000 have been validated in the Encyclopedia of DNA Elements (ENCODE) Project Consortium (GENCODE) [40].

As is in the case of messenger RNAs (mRNAs), lncRNAs are transcribed by RNA polymerase II and then undergo post-transcriptional modifications involving 3′ poly(A) tailing, 5′-end capping and splicing. LncRNA have small open reading frame (ORF) and can be located in the nucleus, or in the cytoplasm [41].

The classification of lncRNAs is complex and is based on several criteria which are not mutually coherent [42]. Based on the genomic context (location, orientation, direction of transcription) relative to protein-coding genes, lncRNAs can be classified into: intron, antisense, intragenic, promoter-associated and enhancer lncRNAs. Intron lncRNAs that are located in the intron of some other transcript. Intergenic lncRNAs are located completely outside of coding genes without any overlap. Of course, some lncRNA are located within exons, or they overlap with other genes. Template strand for the transcription of lncRNAs can be sense or antisense (antisense lncRNAs) and the direction of transcription when lncRNAs and coding genes are transcribed from the same strand can be divergent and convergent. Promoter-associated lncRNAs (PROMPTs) and enhancer RNAs (eRNAs) are transcribed from promoters or enhancer regions [43].

LncRNA exert their function through numerous interactions with DNA, RNA and proteins. Their key role is in the regulation of gene expression, which can be accomplished by acting through chromatin modification, regulation of transcription factors (TFs), or on a post-transcriptional level [44]. According to their molecular mechanisms of action lncRNAs can be classified into four groups: guide, decoy, signaling and scaffold lncRNAs [45,46].

Guide lncRNAs are involved in gene expression regulation through recruitment of ribonucleoprotein (RPN) complex, relocating it to neighboring, or distantly located target genes. In this way via *in cis-* and *in trans-* interactions with chromatin remodeling complex, lncRNA achieve epigenetic regulation of target genes.

Decoy lncRNAs exert their function by sequestering different regulatory molecules, such as transcription factors (TFs), chromatin modifiers or catalytic protein subunits. Thus, by binding to TFs, the lncRNA acts as a “sponge”, removing TFs from the promoter regions of target genes, inhibiting their expression. On the other hand, decoy lncRNAs can act as activators of transcription by binding to TFs leading to their conformational changes and their activation.

Signaling lncRNAs act as a regulatory molecules in a response to diverse stimuli. Their transcription occurs at a specific time and place, depending on the cellular context of the stimuli. One such stimulus is DNA-damage, activating specific expression of LincRNA-p21 and PANDAR [45]. Other signaling lncRNAs are implicated in genetic imprinting by marking certain spaces, times or stages for gene regulation.

Scaffold lncRNA have the ability to bind to multiple effector molecules simultaneously and in that way they serve as a central platform upon which different molecular components can be assembled. To perform this specific function scaffold lncRNAs must possess different binding domains. One such scaffold lncRNA is HOTAIR that adopts a four-module secondary structure in order to interact with poly-comb repressive complex 2 (PRC2) and cause gene repression [47].

Due to the heavy involvement of lncRNAs in important cellular processes, their deregulation has often been associated with different types of cancer [48]. Multiple studies have shown an association of lncRNA expression differences with breast cancer, liver cancer, ovarian cancer, gastric cancer, colorectal cancer, lung cancer, prostate cancer and pancreatic cancer [49].

When it comes to hematological malignancies, the research is limited. Most evidence of specific lncRNA involvement in the etiology, progression, diagnosis, treatment and prognosis of hematological malignancies concerns studies of leukemias. The main pathways through which lncRNAs contribute to the leukemogenic process is through increased oncogene expression, tumor suppressor repression and by increasing cell proliferation [50].

**Figure 2:**
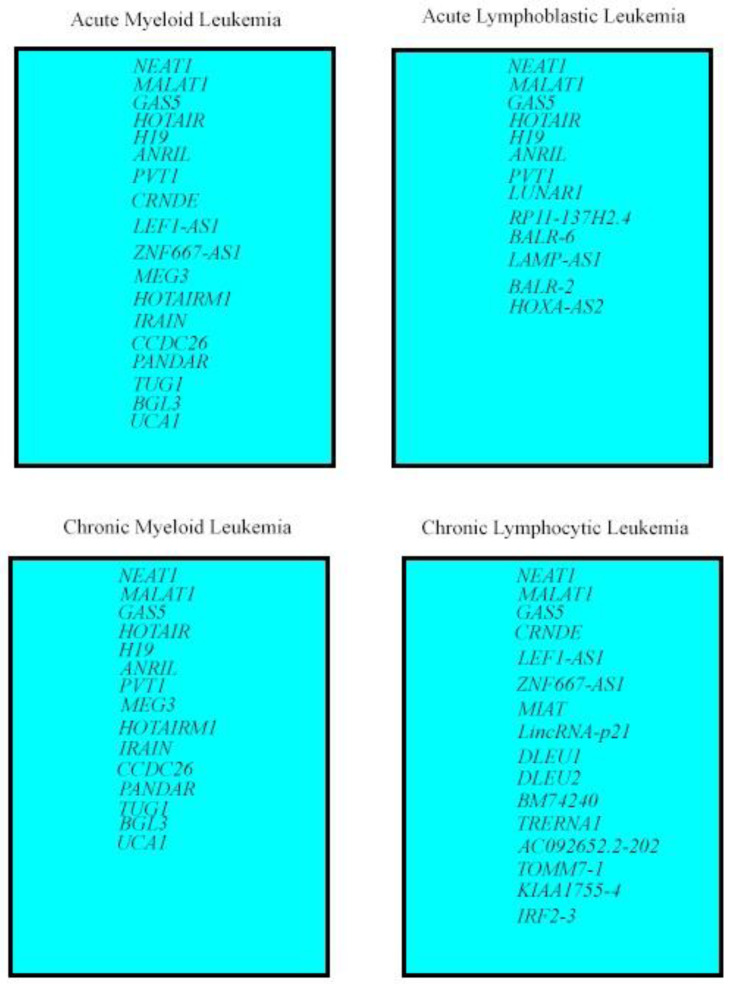
Grouping of important lncRNAs by leukemia type.

The aim of this review is to summarize research data about lncRNAs in different types of leukemia, on their expression pattern, their role in leukemic transformation and disease progression, with an emphasis of how this information can be used in clinical settings, i.e., for diagnostic and prognostic purposes, but also in terms of how particular lncRNAs could be used as potential targets for the application of targeted therapy (Figure 2).

## 3. LncRNAs Deregulated in All Leukemia Types

### 3.1. LncRNA NEAT1

NEAT1 (nuclear enriched abundant transcript 1) is located on chromosome 11q13 and is transcribed into two alternative transcripts generated via 3′- end cleavage. NEAT1_1 (MENε or VINC) is a polyadenylated short transcript (3.7 kb), whereas NEAT1_2 (MENβ) is non-polyadenylated and 23 kb long. Both transcripts are ubiquitously expressed and up-regulated in several tumor cell lines [51]. In association with core proteins PSPC1, PSF and NONO, NEAT1 forms nuclear paraspeckles, highly organized nuclear bodies which involve both NEAT1 transcripts and are formed during NEAT1 transcription at the NEAT1 locus [51,52,53]. NEAT1_2 is exclusively localized in paraspeckles and is essential for their formation, while NEAT1_1 is also located in the nucleoplasm. Novel findings imply that the function of paraspeckles relies on NEAT1_2, whereas NEAT1_1 keeps NEAT1 locus transcriptionally active and enables the switch to NEAT1_2 production in response to stress stimuli [54]. Inhibition of transcription disrupts integrity of paraspeckles and leads to their disintegration [51,55]. NEAT1 is involved in the regulation of gene expression by altering nuclear export of mRNAs with hyper-edited 3′-UTRs. These RNAs, which contain inverted repeats (primarily Alu elements), bind NEAT1 within paraspeckles, leading to their nuclear retention [56]. NEAT1 also sequesters a multitude of proteins from the nucleoplasm into the paraspeckles, some of which regulate different cellular processes such as transcription, splicing and DNA repair [57]. NEAT1 is also a bona fide p53 target [58,59]. It has been demonstrated that activation of p53 by various stress signals that activate DNA damage response, as well as by oncogene-induced replication stress, leads to up-regulation of NEAT1 and formation of paraspeckles. Furthermore, in response to replicative stress, NEAT1 promotes ATR signaling, thus forming a negative regulatory loop that attenuates p53 activity [59]. NEAT1 also acts as decoy by sponging microRNAs and, consequently, modulates the expression of their target genes [60].

In AML, NEAT1 functions as a tumor-suppressor, meaning it is down-regulated in de novo AML. This lncRNA acts as a decoy for miR-23a-3p, which in turn regulates structural maintenance of chromosome 1a (SMC1A). It is assumed that the miR-23a-3p/SMC1A axis is a downstream effector of NEAT1 causing perturbation in the proliferation [61]. A recent study showed that, acting through the NEAT1/miR-338-3p/CREBRF axis, this lncRNA could contribute to AML progression [62]. CREB3 regulatory factor (CREBRF) is a highly conserved protein with known anti-cancer action that is down-regulated in AML patients, with significant role in migration, invasion and apoptosis of AML cells.

Among different types of AML, low expression of NEAT1 is most prominent in the APL (AML-M3) subgroup of patients. Acute pro-myleocytic leukemia is characterized by the presence of the PML-RARa fusion onco-protein, which arises as a result of t(15;17) and represents the primary transforming event, causing a blockage in promyelocytic differentiation process. It was shown that the PML-RARa fusion protein inhibits NEAT1 expression, but the treatment with all-trans retinoic acid (ATRA) can restore its expression and promote differentiation to granulocytes [62].

Furthermore, it has been shown that *NEAT1* expression is down-regulated in patients with AML and its expression levels are in correlation with *PTEN*, a known tumor suppressor [63].

In childhood ALL, multidrug resistance is generated by a higher expression of *NEAT1*, which competes for a miRNA seed region of miR-335-3p, a regulator of expression of a transporter, ABCA3, whose overexpression contributes to multidrug resistance [64]. These results are in contrast with studies on leukemia cell lines, where overexpression of *NEAT1* lessened multidrug resistance [65].

NEAT1 was found to be up-regulated in primary CML cells. The mechanism of action of NEAT1 in CML is closely associated with the BCR-ABL fusion onco-protein, i.e., to the regulation of signaling pathways following the constitutive tyrosine-kinase activation [63]. It was found that the repression of NEAT1 is under direct control of c-MYC that binds to the NEAT1 promoter. c-MYC represents a common regulator of BCR-ABL downstream signaling pathways [66]. Transcriptional repression of NEAT1 leads to deregulation of the paraspeckle protein splicing factor proline/glutamine-rich (SFPQ) component of paraspeckles that is necessary for NEAT1-induced apoptosis. In imatinib-treated CML cells, inhibition of BCR-ABL tyrosine-kinase activity restores NEAT1 expression and causes apoptosis via the BCR-ABL/c-MYC/NEAT1/SFPQ axis [65].

Experiments on primary CLL cells showed that both DNA damage and non-genotoxic activation of p53 response leads to up-regulation of NEAT1 (only in the TP53^wt^ setting). Impaired NEAT1 induction in cells with 11q deletion, correlation of p21 and NEAT1 expression levels and loss of cell viability were also demonstrated [58]. In a recent study conducted on a cohort of newly diagnosed Binet A CLL patients, the expression of global NEAT1 and NEAT1_2 was found not to be statistically different compared to normal B cells, but was higher than in other hematological malignancies investigated (with the exception of multiple myeloma). In addition, CLL cells expressed the highest amount of NEAT1_2 isoform in comparison to other cell types. Although global NEAT1 expression showed no association with IGHV mutational status, cytogenetic abnormalities, NOTCH1 and TP53 mutational status and clinical outcome, NEAT1_2 isoform expression was significantly higher in IGHV mutated CLL, as well as in cases with 13q deletion or without cytogenetic aberrations. Low NEAT1_2 level was also associated with shorter time to first treatment, although not independently from other prognostic factors [67].

### 3.2. LncRNA MALAT1

MALAT1 (metastasis-associated lung adenocarcinoma transcript 1) or NEAT2 is lncRNA located on chromosome 11q13, 60 kb downstream of the NEAT1 locus. Like NEAT1, MALAT1 is also a single-exon gene, transcribed into a 8.7 kb long primary transcript which is processed by cleavage of a tRNA-like small ncRNA from its 3′ end. The resulting mature MALAT1 lncRNA is stabilized by a blunt-ended triple helical structure at the 3′ end and is retained in the nucleus within the nuclear speckles [68,69]. Nuclear speckles are nuclear bodies that accumulate splicing factors, as well as RNA processing and export factors. As opposed to NEAT1, MALAT1 is not a structural component of nuclear speckles; rather it is recruited to speckles when they are already formed and upon initiation of transcription [68,70]. MALAT1 regulates alternative splicing through direct interaction with serine/arginine-rich splicing factors leading to their localization to nuclear speckles and recruitment to the site of transcription. In addition, MALAT1 modulates the cellular levels of serine/arginine-rich proteins, as well as their phosphorylation [70]. MALAT1 also affects transcription via: (1) interaction with transcriptional factors and their recruitment to promoters of target genes; (2) interaction with components of poly-comb repressive complex 2 (PRC2), thus inducing trimethylation of histone H3 at lysine 27 (H3K27me3) and, consequently, transcriptional repression; (3) sequestering miRNAs through miRNA-responsive elements located in its sequence, thus activating the expression of their targets [71]. MALAT1 exerts a pro-proliferation function and is up-regulated in many cancers. During normal cell cycle progression, MALAT1 levels are tightly regulated, with low levels during G1 and G2 and high levels during G1/S and mitosis. It has been demonstrated that MALAT1 modulates the expression of genes involved in cell cycle progression and/or their pre-mRNA processing. Activation of p53 and its target genes in MALAT1-depleted cells pointed to p53 as a major downstream effector of MALAT1 activity [72].

LncRNA MALAT1 was found to be overexpressed in de novo AML patients, compared to healthy controls and patients in complete remission (CR) [73]. MALAT1 performs its oncogenic function by acting as a decoy for miR-96 which is involved in the process of proliferation and whose decreased expression is a poor prognostic marker in AML [74]. Similar to CML, in AML overexpression of MALAT1 is associated with the resistance to therapy. Down-regulation of MALAT1 increases the efficiency of cytarabine (Ara-C) therapy through the MALAT1/miR-96 axis [75].

In ALL, MALAT1 acts as a sponge for miR-205, thus regulating PTK7 expression, a known oncogene. MALAT1 was up-regulated in ALL samples and it has been shown that its overexpression promoted proliferation and inhibited apoptosis [76]. MALAT1, like NEAT1, acts as a sponge for miR-335-3p, thus contributing to chemoresistance in childhood ALL [64].

In CML, MALAT1 also has an oncogenic function, but its overexpression is primarily associated with resistance to imatinib therapy. In CML, MALAT1 acts as a decoy for miR-328 that is involved in cell-cycle regulation. Therefore, down-regulation of MALAT1 causes cell-cycle arrest and suspension of proliferation through the MALAT1/miR-328 axis. More importantly, MALAT1/miR-328 pathway can be targeted in an attempt to increase imatinib sensitivity [77].

In CLL, a significantly higher expression of MALAT1 in peripheral blood mononuclear cells was observed when compared to healthy controls, implying its involvement in tumorigenic processes. However, MALAT1 levels were not associated with the prognostic groups based on cytogenetic aberrations [78].

### 3.3. LncRNA GAS5

Growth arrest-specific transcript 5 (GAS5) lncRNA is located at chromosome 1q25, encoding a 650 bp long transcript containing 12 exons [79,80]. The exons encode a few splice variants of GAS5, but due to the presence of a stop codon, none of them produce proteins and the transcripts are degraded through the nonsense mediated decay (NMD) pathway [81]. GAS5 is down-regulated in many types of cancer indicating a tumor-suppressor function of this lncRNA [81]. GAS5 performs a tumor-suppressor through various mechanisms. As a signal molecule, GAS5 directly participates in the regulation of the p53 signaling pathway. In that way, decreased expression of GAS5 is associated with cell-cycle arrest via increased p53 expression [82]. GAS5 can function as a decoy, a molecular “sponge”, binding directly to different target RNAs or proteins [83].

There are only few studies examining GAS5 in AML, one of them examining GAS5 gene polymorphisms and their impact on AML prognosis [84]., Only two studies were investigating expression level of GAS5 in AML samples and based on their findings GAS5 expression level in AML patients was lower compared to healthy controls [82,85]. This suggests that in AML GAS5 has a tumor-suppressive function and it was also found that lower expression of GAS5 was associated with inferior outcome among younger AML-NK patients [85]. As is the case with CLL, in AML GAS5 exerts its tumor-suppressor function, acting as a decoy for miR-222 [85].

In ALL, *GAS5* has shown higher expression in patients who poorly responded to GC treatment in the remission-induction phase of childhood ALL treatment [86]. Furthermore, *GAS5* overexpression has shown significant association with a higher risk of short-term relapse and poor treatment outcome [87].

LncRNA GAS5 expression was reported to be decreased in mononuclear cells of CLL patients in comparison to healthy controls, implying its tumor suppressor function in CLL. Overexpression and knockdown experiments performed on Raji cells showed that lncRNA GAS5 overexpression reduces cell proliferation, promotes apoptosis, induces cell cycle arrest at G1 phase and decreases cell invasion. Dual luciferase reporter assay identified miR-222 as a direct target of lncRNA GAS5. This was in line with the observed overexpression of miR-222 in CLL samples and the negative correlation between the levels of lncRNA GAS5 and miR-222 in both CLL samples and Raji cells overexpressing lncRNA GAS5 [88]. Given that miR-222 in CLL cells down-regulates the expression of p27, which prevents cell cycle progression, low level of lncRNA GAS5 in CLL could promote cell proliferation by inhibiting p27 [88,89].

Details on the role of lncRNAs deregulated in all leukemia types are found in Table 1.

## 4. LncRNA Deregulated in Myeloid Leukemias and ALL

### 4.1. LncRNA HOTAIR

LncRNA, HOX transcript antisense intergenic RNA (HOTAIR) is expressed from the HOXC locus on chromosome 12q13.31 and due to its increased expression in many cancers it is believed to play an oncogenic role. HOTAIR interacts with epigenetic regulators such as poly-comb repressive complex 2 (PRC2) and lysine-specific demethylase 1 (LSD1), causing chromatin reprograming and modulating gene transcription. This lncRNA acts as a scaffold bringing together different molecules inducing gene repression [47].

Increased expression of HOTAIR in AML causes uncontrolled proliferation of leukemia stem cells (LSCs). Namely, HOTAIR regulates self-renewal of LSCs through EZH2-dependent epigenetic silencing of the tumor suppressor p15^INK4b^ gene (cyclin-dependent kinase inhibitor 2B –CDKN2B gene) [116]. HOTAIR also performs its oncogenic role by acting as a decoy for tumor-suppressor miR-193a. High expression of miR-193a detected in AML patients inhibits proliferation and induces apoptosis in leukemia cells by modulating c-KIT expression [117]. Recently, another mechanism by which HOTAIR could contribute to the leukemogenic process was found and it involves silencing of the HOXA5 tumor-suppressor gene through increased methylation of its promoter [156].

In AML HOTAIR can be used as a molecular biomarker for poor prognosis, because its overexpression is associated with adverse clinical characteristics such as a higher number of blasts, shorter DFS and OS [117,118,157]. High expression of HOTAIR was associated with increased expression of multidrug resistance protein 1 (MRP1) in the imatinib resistant CML patents. It was shown that in the K562 cell line, HOTAIR mediates PI3K/Akt signaling pathway and causes resistance [119]. As previous research conducted on solid tumors showed that high HOTAIR expression was associated with resistance to anthracycline therapy (primarily doxorubicin), it can be assumed that a similar mechanism for acquired chemoresistance can occur in AML patients treated with these drugs [158].

HOTAIR expression in B-ALL patients was shown to be significantly higher [159].

### 4.2. LncRNA H19

H19 imprinted maternally expressed transcript is located at chromosome 11p15.5 near the insulin-like growth factor 2 (IGF2) gene and encodes an approximately 2.3-kb lncRNA located in the cytoplasm that can act as a tumor-suppressor or oncogene [120,160]. In normal hematopoiesis, H19 is responsible for maintaining hematopoietic stem cell (HSC) quiescence and regulation of long-term homeostasis of HSCs [161].

It was shown that H19 high-expressing AML patients had shorter OS and lower CR rate, indicating that H19 expression can be used as a prognostic marker [111]. H19 performs its oncogenic role through different mechanisms. It can regulate cell proliferation acting as a decoy for miR-19a and miR-19b and by directly targeting the ID2 gene [112]. Through another target, miR-29a-3p, H19 can influence not only cell growth, but also apoptosis via the Wnt/β-catenin pathway [113]. In addition, H19 may be involved in telomerase activity. In in vitro studies done on the NB4 cell line (acute promyelocytic leukemia (APL) -AML-M3 cell line), ATRA (all-trans retinoic acid) induces overexpression of H19 and causes the decrease of telomerase activity necessary for tumor growth [162].

H19 is also highly expressed in primary CML cells and it is presumed to be involved in BCR-ABL-mediated oncogenesis. Namely, it was shown that H19 is up-regulated through c-MYC and required for leukemic process in CML. The same authors also indicated that inhibition of H19 expression makes CML cells more susceptible to imatinib and halts BCR-ABL-induced proliferation [97]. The presence of H19 overexpression is also implicated in the tendency towards disease progression and poor prognosis in CML patients [114].

In ALL, H19 has been shown to be oncogenic and significantly increased in expression in newly diagnosed pediatric ALL patients. It also acts as a sponge for miR-326, a confirmed tumor suppressor. The lower expression of miR-326 contributes to greater *BCL-2* expression [115].

### 4.3. LncRNA ANRIL (CDKN2B-AS1)

The lncRNA Antisense Non-coding RNA in the INK4 Locus (ANRIL), also known as CDKN2B-AS1 is antisense lncRNA from the INK4b-ARF-INK4a gene cluster on chromosome 9p21.3. The INK4b-ARF-INK4a locus encodes p14^ARF^, p15^INK4b^ and p16^INK4a^ tumor suppressor genes that are involved in key cellular processes such as apoptosis, self-renewal and senescence of hematopoietic stem cells [163]. P14^ARF^, p15^INK4b^ and p16^INK4a^ are important cyclin-dependent kinase (CDK) inhibitors [164]. ANRIL suppresses expression of these CDK-inhibitors by recruiting poly-comb repressive complexes PRC1 and PRC2 to the INK4b-ARF-INK4a locus, which results in gene silencing and, further, to cell-cycle arrest and block in differentiation and apoptosis [91].

In AML, ANRIL has an oncogenic role and therefore its expression is increased and associated with a poor prognosis [92]. In AML, ANRIL is also essential for maintenance of a leukemic state in the way that it regulates glucose metabolism through targeting ADIPOR1 (adiponectin receptor) and its downstream factors adenosine monophosphate-activated protein kinase (AMPK)/sirtuin 1 (SIRT1) [93].

Concerning ALL, ANRIL is up-regulated in T-ALL [165]. In knockdowns, T-ALL cells showed lower viability, migratory and invasion capabilities. The lncRNA also acts as a sponge for miR-7-5p, downregulating it and, thus, upregulating *TCF4*, known to be overexpressed in numerous tumors and leukemic cell lines [94]. ANRIL has also shown greater expression in B-ALL patients, showing lower expression upon complete remission and an increase in the level of expression on relapse [166].

### 4.4. LncRNA PVT1

Plasmacytoma variant translocation 1 (PVT1) is an intragenic lncRNA encoded by the PVT1 gene located at chromosome 8q24. This region is frequently disrupted in numerous cancers either by rearrangements or by amplifications [167]. In the close proximity to PVT1 gene, a well-known oncogene, c-MYC, is located and moreover, several c-MYC enhancer elements are positioned within the PTV1 gene locus [168]. The existence of a positive feedback loop between these two genes was established. Namely, PVT1 inhibits degradation of c-MYC protein, which in turn increases the expression of the PTV1 gene by binding to the c-MYC gene promoter [169].

High expression of PTV1 was detected in patients with acute promyelocytic leukemia (APL) and experiments done on NB4 (human APL cell line) have shown that knockdown of c-MYC causes decrease in *PVT1* expression and knockdown of *PVT1* results in the decrease of c-MYC protein, causing inhibition of cell proliferation. Treatment with ATRA causes repression in *PVT1* expression and impairs proliferation [141,169]. High expression of PVT1 is also detected among AML patients with recurrent t(8;21) causing lower OS [142]. In in vitro studies it was shown that inhibition of PVT1 expression induces apoptosis and necrosis in AML cell line, so it was assumed that PVT1 performs its oncogenic role by regulating apoptosis [143].

In ALL, one of the mechanisms of *PVT1* effect is the sponging of miR-486-5p, a micro RNA whose downregulation increased cell viability and decreased apoptosis of ALL cells [170]. Long non-coding RNA (lncRNA) plasmacytoma variant translocation 1 gene (PVT1) modulates the proliferation and apoptosis of acute lymphoblastic leukemia cells by sponging miR-486-5p [170].

Details of the role of lncRNAs deregulated in myeloid leukemias and ALL are found in Table 1.

## 5. LncRNAs Deregulated in AML and CLL

### 5.1. LncRNA CRNDE

CRNDE (colorectal neoplasia differentially expressed; also known as lincIRX5) locus is located on chromosome 16q12.2, next to IRX5 gene. It contains a total of six exons, encoding 14 different splice variants. Reduced expression of CRNDE in CLL cells was shown to positively correlate with the expression of the neighboring IRX5 gene, involved in apoptosis and proliferation, suggesting *in cis* coregulation [171]. Experiments on CLL cell lines revealed that both overexpression and demethylation of CRNDE, leading to its up-regulation, repressed cell proliferation and induced apoptosis. This effect was mediated by CRNDE function as a decoy by binding to miR-28 causing its repression, which controls its downstream target NDRG2 [102].

In AML CRNDE has an oncogenic function. High expression of CRNDE is associated with lower CR and shorter disease-free survival (DFS) and over-all survival (OS), and therefore can be used as a molecular marker predicting inferior outcome [103,104]. Especially high expression level was detected among APL and NPM1-mutated AML patients, promoting proliferation of leukemic cells and blocking myeloid differentiation. It was shown that CRNDE located in the cytoplasm of APL cells functions as a decoy and binds miR-181 [105]. This miR-181 regulates one its downstream target genes NOTCH2 that is, like CRNDE, highly expressed in AML. As an oncogenic signaling pathway, NOTCH is involved in differentiation, proliferation and apoptosis of leukemic cells [172].

Apart from this CRNDE/miR-181/NOTCH2 axis, CRNDE elicits its oncogenic potential by binding to miR-136-5p inhibiting its expression. Down-regulation of this tumor suppressor miRNA in turn, up-regulates one of the miR-136-5p targets, MCM5 (mini-chromosome maintenance protein 5) gene and modulates cell progression [106].

High expression of CRNDE was also detected among AML patients resistant to adriamycin (ADR)-based chemotherapy. Up-regulation of CRNDE expression was significantly associated with increased expression of the multidrug resistance protein 1 (MDR1) gene, with is well known role in drug resistance. It was shown that knockdown of CRNDE results in suppressed proliferation and multidrug resistance in ADR-resistant AML cells through inhibition the Wnt/ß-catenin pathway [107].

In CLL, deregulation of CRNDE was detected through comparison analyses of the DNA methylation profile between CLL cells and normal B cells. Apart from CRNDE, which was found to be hyper-methylated, lncRNA AC012065.7 was found to be hypo-methylated at their promoters. The methylation levels of both genes were inversely correlated to their expression and were associated with shorter overall survival [102]. Up-regulation of AC012065.7 (chromosome 2p24), due to hypo-methylation, was found to be positively correlated with the expression of its neighboring gene GDF7, which regulates diverse processes in growth, repair and embryonic development [171].

### 5.2. LncRNA LEF1-AS1

LncRNA LEF1-AS1 is an antisense long non-coding RNA encoded in the lymphoid enhancer-binding factor 1 (LEF1) locus (chromosome 4q25) and localized in the nucleus.

In AML lncRNA LEF1-A1 is down-regulated compared to healthy controls. Induced over-expression of LEF1-AS1 in a myeloid cell line and in patient’s mononuclear cells caused reduced proliferation through increased expression of CDKN1A (p21) and CDKN1B (p27) tumor suppressors [173].

In a cohort of newly diagnosed CLL patients, LEF1-AS1 was found to be up-regulated in comparison to healthy controls, but the association with baseline patients’ characteristics and established prognostic markers was not observed [128]. Overexpression of LEF1-AS1 in CLL cell lines via lentiviral transduction resulted in increased survival and inhibition of apoptosis, implying its oncogenic role in CLL. In addition, correlation between lncRNA LEF1-AS1 and LEF1 expression was demonstrated, as well as specific binding of lncRNA LEF1-AS1 and LEF1 protein. Thus, LEF1-AS1 may exert its oncogenic potential through regulation of target LEF1, whose high expression in CLL is associated with poor prognosis [127,128].

### 5.3. LncRNA ZNF667-AS1

Zinc finger protein 667-antisense RNA 1 (lncRNA ZNF667-AS1) is located at chromosome 19q13.43, within a cluster of zinc finger genes [174]. As is the case with CLL, overexpression of this lncRNA in AML patients is associated with poor outcome [153,175]. Based on these findings, it can be assumed that ZNF667-AS1 could have an oncogenic role in AML. It was found that ZNF667-AS1 acts as a molecular sponge for miR-206 with known tumor-suppressor function in pediatric AML patients [154]. Down-regulation of ZNF667-AS1 reduces proliferation and invasion of leukemic cells, acting through miR-206/A-kinase anchoring protein 13 (AKAP13) axis, or miR-206/Cyclin D1 axis [153,154]. It is important to note that TCGA data analysis showed that expression of ZNF667-AS1 is down-regulated in APL (AML-M3), implying its tumor-suppressor function in this subtype of AML, emphasizing the tissue specific function of lncRNAs [110].

Details on the role of lncRNAs deregulated in AML and CLL are found in Table 1.

## 6. LncRNAs Deregulated in Myeloid Leukemias

### 6.1. LncRNA MEG3

Maternally expressed 3 (MEG3) is located on chromosome 14q32, consisting of 10 exons that encode an approximately 1.6-Kb long transcript, but is prone to alternative splicing. In hematological malignancies MEG3 has a tumor-suppressor function acting through the p53 and TGF-β pathways [155].

In AML, it was shown that increased expression of MEG3 is a reliable molecular marker of favorable prognosis, both in pediatric and adult patients. In adult de novo AML patients, high MEG3 expression was independent predictor for longer DFS and OS [128], while in pediatric de novo AML patients increased MEG expression was associated with prolonged OS [133,134]. High MEG3 expression in AML patients after induction therapy was also associated with better DFS and OS, indicating that it could be used as a marker for the prediction of therapy response.

Indeed, in CML patients, down-regulation of MEG3 was associated with the resistance to imatinib. Imatinib resistant cells were characterized by low expression level of MEG3 and high expression level of its target, miR-21 [135]. Decrease in MEG3 expression was accompanied by the progression of the disease from chronic phase to the CML in blast crisis and all of this was caused by the advanced hyper-methylation of MEG3 promoter [137]. Inhibition of cell proliferation and metastasis and prompted apoptosis of resistant CML cells could be initiated through forced expression of MEG3 interacting with its mediators miR-184 and miR-147 [176,177].

LncRNA MEG3 is implicated in the occurrence of the drug resistance in AML through the MEG3/miR-155/ALG9 axis. Acting as a decoy for miR-155, MEG3 alters the level of ALG9 protein, whose expression is known to be down-regulated in resistant cells [136,178]. ALG glycosyltransferase 9 (ALG9) is involved in the glycosylation process, one of the important mechanisms of post-transcriptional modification. Overexpression of MEG3 causes G0/G1 cell-cycle arrest and obstructs proliferation, but also induces apoptosis through p53-dependent signaling pathway inhibiting the leukemogenesis. Up-regulation of MEG3 increases ALG9 expression and restoring drug sensitivity in AML cells [136,178].

### 6.2. LncRNA HOTAIRM1

HOXA transcript antisense RNA, myeloid-specific 1 (HOTAIRM1) is located at chromosome 7p15.2, between the HOXA1 and HOXA2 gene clusters. This intragenic lncRNA is expressed in the myeloid lineage and is involved in regulation of the granulocytic differentiation process in normal hematopoiesis [179]. Some of the target genes for HOTAIR such as HOXA1, HOXA4, CD11b and CD18 are required for granulopoiesis. Experiments done on NB4 human acute pro-myelocytic leukemia cell line showed that HOTAIRM1 expression is up-regulated during ATRA-induced granulocytic differentiation promoting cell-cycle progression [121].

HOTAIRM1 can act as a decoy for miR-20a, miR-106b and miR-125b which are very important in autophagy regulation. Autophagy is a process that is necessary for proper differentiation and for maintaining cellular homeostasis. Therefore, through binding with miR-20a, miR-106b and miR-125b, HOTAIRM1 diverts them from their target genes involved in autophagy pathway (ULK1, E2F1 and DRAM2) [124]. In another study HOTAIRM1 targets miR-148b and leads to progression of AML [123].

HOTAIRM1 is highly expressed in AML patients and it can be used as a molecular marker for poor prognosis because it is associated with shorter DFS and OS [180]. Overexpression of HOTAIRM1 was associated with intermediate cytogenetic risk AML patients and especially with the presence of NPM1 gene mutations [122]. More precisely, mutant NPM1 caused increased expression of HOTAIRM1 via KLF5-dependent transcriptional regulation. Same authors detected that HOTAIRM1 in nucleus acts as a scaffold for formation of the MDM2-EGR1 complex and induces degradation of EGR1 transcriptional factor, while HOTAIRM1 in cytoplasm acts as a sponge for miR-152-3p increasing expression of ULK3 kinase and induces autophagy [122]. All of the above indicates that this lncRNA is a promising target for the design and application of specific therapy.

### 6.3. LncRNA IRAIN

Insulin-like growth factor 1 receptor (IGF1R) antisense imprinted non-protein coding RNA, or IRAIN, is a lncRNA located at chromosome 15q26.3 within the GF1R locus and it is transcribed into a 5.4kb product [181]. IRAIN is transcribed antisense from IGF1R that is a key component of the PI3K/Akt signaling pathway, known to be constitutively active in AML, promoting proliferation process in leukemic cells [182]. In direct interaction with chromatin DNA in the IGF1R enhancer and promoter, IRAIN participates in the formation of an enhancer-promoter loop, or intra-chromosomal loop between the IGF1R enhancer and promoter [183]. Still, functional implications of IRAIN’s role in this intra-chromosomal loop formation are not known.

In AML, low expression of IRAIN is associated with poor prognosis, suggesting that IRAIN has a tumor-suppressor function. It is reported that in non-APL acute myeloid leukemia patients, diminished expression of IRAIN results not only in shorter OS but also in a refractory response to chemotherapy [126].

### 6.4. LncRNA CCDC26

Another lncRNA is located at chromosomal locus 8q24 is CCDC26 (coiled-coil domain-containing 26). It is overexpressed in AML patients and it is associated with poor prognosis and thus can be used as a molecular biomarker in risk stratification of AML [99,100]. CCDC26 performs its oncogenic function in AML by regulation of c-KIT—tyrosine kinase receptor expression and up-regulation of this lncRNA increases cell growth [101].

### 6.5. LncRNA PANDAR

LncRNA promoter of CDKN1A antisense DNA damage activated RNA (PANDAR) is also over expressed in AML patients and associated with lower CR rate and shortened survival [184]. PANDAR is involved in the DNA-damage response, its induction is p53 dependent. In AML overexpression of PANDAR promotes cell survival by blocking the expression of proapoptotic genes through interaction with NF-YA transcription factor preventing it from binding to target gene promoters [139].

### 6.6. LncRNA TUG1

Taurine up-regulated 1 (TUG1) lncRNA is overexpressed in AML and it is associated with a number of clinical and molecular characteristics such as higher WBC count, presence of FLT3-ITD mutation and mono-somal karyotype related with high risk disease. Therefore it could be used as a molecular marker for poor prognosis [140].

In in vitro studies it was shown that TUG1 increases cell proliferation and suppresses apoptosis by targeting aurora kinase A (AURKA) [145]. Recent study also provided evidence that TUG1 is involved in promoting chemoresistance to adriamycin (ADR) therapy through EZH2-mediated epigenetic silencing of miR-34a [185]. TUG1 plays its oncogenic role acting as a decoy for tumor-suppressor miR-221-3p, promoting cell viability and inhibiting apoptosis [147].

### 6.7. LncRNA BGL3

Beta globin locus 3 (BGL3) is a lncRNA located at chromosome 11p15.4, involved in BCR-ABL-mediated cell transformation in CML and ALL. BCR-ABL inhibits BGL3 expression via MYC-dependent DNA methylation and loss of BCR-ABL results in the increase of BGL3, decrease of MYC expression and sensitization of leukemia cells towards imatinib-induced apoptosis, suggesting that BGL3 acts as a tumor-suppressor in CML [146]. In addition, BGL3 has a decoy function by binding to miR-17, miR-20a, miR-20b, miR-93, miR-106a and miR-106b altering the function of PTEN (phosphatase and tensin homolog) tumor-suppressor [146].

### 6.8. LncRNA UCA1

Urothelial Carcinoma-associated 1 (UCA1) is a lncRNA located on chromosome 19p13 [186]. UCA1 is capable of interacting with several different epigenetic regulators, among others p27^Kip1^, a well-known tumor-suppressor involved in the suppression of cyclin–dependent kinase [187,188]. UCA1 was found to be up-regulated in AML, especially among patients carrying mutation in CCAAT/enhancer-binding protein-A (CEBPA) transcriptional factor. Overexpression of UCA1 in CEPBA-mutated patients induces cell proliferation through inhibition of the p27 ^Kip1^ cell-cycle regulator [189].

By sponging miR-125, UCA1 increases expression of hexokinase-2 (HK2), a key enzyme involved in glycolysis. UCA1 inhibits glycolysis and is involved in the presence of chemoresistance in AML acting through the UCA1/miR-125a/HK2 axis. Knockdown of UCA1 expression in adriamycin (ADR)-resistant AML cells resulted in the reversion of chemosensitivity [148]. UCA1 also targets miR-126 thus activating JAK/STAT and PI3K/AKT signaling pathway and providing viability, migration and invasion of AML cells [150]. A number of new studies detected that UCA1 targets other miRNAs such as miR-204, miR96-5p and miR296-3p and through these interactions it exerts its oncogenic potential in AML [149,190,191].

In CML, UCA1 mediates resistance to imatinib therapy. Overexpressed UCA1 binds miR-16, stimulating expression of MDR1 and enhancing imatinib resistance in CML-cells [192]. This finding indicates that UCA1 is a potential target for reversal of drug resistance in BCR/ABL-positive CML patients.

Details on the role of lncRNAs deregulated in myeloid leukemias are found in Table 1.

## 7. LncRNAs Deregulated in ALL

### 7.1. Lnc RNA LUNAR1

Leukemia-induced non-coding activator RNA 1 (LUNAR1) is a lncRNA located on the chromosome 15q26.3, where the locus for *IGF1R* is located [151]. LUNAR1 is a *cis*-regulator which regulates the expression of *IGF1R*. It has been shown that LUNAR1 is downregulated in Notch inhibition and upregulated in T-ALL. Upregulation of LUNAR1 was associated with a poor prognosis in pediatric T-ALL [131].

### 7.2. Lnc RNA RP11-137H2.4

This lncRNA is located at chr10:80529597-80535942 and is a *TSPAN14* antisense RNA. It modulates the expression of components of NRAS/BRAF/NF-kB MAPK cascade and cell cycle pathways. Silencing this lncRNA removed glucocorticoid resistance in pre-B pediatric ALL cell lines [132].

### 7.3. Linc RNA BALR-6

B-Cell acute lymphoblastic leukemia associated long RNA 6 (BALR-6) is a lincRNA located at 3p24.3, a block between *SATB1* and *TBC1D5*. It regulates the expression of *SP1* and *CREB1*, whose products have significant effects on cell growth, proliferation and survival. The largest BALR-6 expression was detected in cell lines with MLL rearrangements [95]. Research has shown that BALR-6 knockdowns inhibit proliferation in B-ALL cell lines, its constitutive expression is important for cell survival and proliferation in cell lines, while its overexpression promotes hemato-poetic progenitor proliferation in patients [96].

### 7.4. LncRNA LAMP-AS1

Lysosomal-associated membrane protein 5 antisense 1 (LAMP5-AS1) lncRNA is located on chromosome 20p12.2, where it is highly expressed and correlated with the adjacent gene, *LAMP5*. It is genetically regulated by H3K79 methylation [193]. This lncRNA has been shown to be specifically overexpressed in MLL rearrangement positive childhood leukemias and higher expression level patients had reduced 5-year leukemia-free survival [194].

### 7.5. LncRNA BALR-2

BALR-2 is similar to BALR-6. It was found to be upregulated in B-ALL pediatric patients that were glucocorticoid resistant and higher BALR-2 expression was associated with poor overall survival. Knockdown of this lncRNA led to growth inhibition and promoted apoptosis [95].

### 7.6. LncRNA HOXA-AS2

HOXA cluster antisense RNA 2 (HOXA-AS2) is an antisense lncRNA, located at chr7:27113936-27134302. This lncRNA was highly expressed in glucocorticoid resistant pediatric ALL patients. In the presence of dexamethasone, HOXA-AS2 promoted proliferation and reduced apoptosis of glucocorticoid resistant cells [195].

Details on the role of lncRNAs deregulated in ALL are found in Table 1.

## 8. LncRNAs Deregulated in CLL

### 8.1. LncRNA MIAT

MIAT (myocardial infarction associated transcript; also known as GOMAFU, LINC00066, RNCR2) is lncRNA encoded by 30,051 bp long intergenic region located on 22q12.1. The MIAT gene consists of five exons and is transcribed into approximately 10 kb long RNA which is polyadenylated and spliced, giving rise to four splice variants [125]. Despite having protein-coding mRNA characteristics, MIAT RNA escapes nuclear export and accumulates in the nucleus. It exerts no association with chromatin, like other nuclear-retained lncRNAs, but is associated with the nuclear matrix forming nuclear bodies scattered throughout the nucleus [55,196]. MIAT RNA contains multiple tandem UACUAAC repeats which are recognized by splicing factor 1 (SF1) [196]. This suggests that MIAT modulates local concentrations of SF1, thus affecting the efficiency of splicing events and, consequently, gene expression.

Although MIAT expression is restricted to several subsets of neurons, MIAT up-regulation has been detected in a number of mature B cell leukemia/lymphoma cell lines and in primary CLL cells [55,196,197]. In addition, it has been demonstrated that high expression of MIAT in peripheral blood mononuclear cells of CLL patients is associated with adverse prognostic markers such as high and intermediate risk cytogenetics (del17p, del11q and trisomy 12) and unmutated IGHV status, as well as with shorter overall survival [197]. Knockdown experiments on a diffuse large B cell lymphoma (DLBCL) cell line indicated that in mature malignant B cells MIAT regulates the expression of its own transcriptional regulator OCT4, forming a positive feedback loop. Furthermore, suppression of MIAT or OCT4 induced apoptosis and led to cell growth inhibition [197]. These results imply that MIAT upregulation may be involved in apoptotic resistance of CLL B lymphocytes and, at least in part, accounts for the observed association with the aggressiveness of the disease.

### 8.2. LincRNA-p21

LincRNA-p21 gene locus is located on chromosome17, around 15 kb upstream of the CDKN1A (p21) gene, which mediates cell-cycle arrest as a part of p53 pathway and its direct transcriptional target. LincRNA-p21 is transcribed in the opposite orientation from the CDKN1A gene into 3.1 kb transcript containing two exons [138]. It has been demonstrated that, upon induction of DNA damage in different tumor cell lines, including lymphoma, lincRNA-p21 promoter is bound by p53 resulting in transcriptional activation. On the other hand, lincRNA-p21 directly interacts with MDM2 and up-regulates MDM2-p53 interaction, thus forming a feedback loop which modulates p53 activity [129]. In addition, experiments with RNA interference-mediated knockdown of lincRNA-p21 and p53 identified many co-reregulated genes and showed that lincRNA-p21 acts as a global repressor of genes involved in p53-dependent transcriptional response. Both lincRNA-p21 and p53 knockdowns caused the repression of proapoptotic and de-repression of antiapoptotic genes, suggesting that lincRNA-p21 plays a role in the p53-dependent induction of apoptosis. In these experiments, however, lincRNA-p21, in contrast to p53, did not substantially affect transcription of CDKN1A and other cell-cycle regulators and did not contribute to cell-cycle arrest following DNA damage [138]. The mechanism of lincRNA-p21-mediated transcriptional repression involves its direct interaction with heterogeneous nuclear ribonucleoprotein K (hnRNP-K), a component of repressor complexes that operate in p53 pathway, through conserved 780 nt long 5′ region. The lincRNA-p21 modulates hnRNP-K localization and enables its binding to the promoters of genes that are co-repressed by p53 and lincRNA-p21 [138]. Besides in transaction of lincRNA-p21, it has been demonstrated that it also operates *in cis* and activates p53-mediated expression of the neighboring Cdkn1a gene [130,198]. Finally, lincRNA-p21 is involved in heterochromatin regulation through the association with H3K9 methyltransferase SETDB1 and the maintenance of DNA methyltransferase DNMT1, which are both mediated by hnRNP-K, thus sustaining H3K9me3 and/or CpG methylation of pluripotency gene promoters [199].

Analyses of lincRNA-p21 expression in primary CLL cells in the context of DNA damage revealed its induction in a p53-dependent manner: the strongest induction was observed in TP53^wt^ cells, but was impaired in CLL cells with 11q deletion (which encompasses ATM gene) and TP53^del/mut^. A positive correlation of lincRNA-p21 with the induction of p21 was also observed, as well as decrease in cell viability, again only in TP53^wt^ cells. These results were confirmed on a set of Burkitt’s lymphoma cell lines, suggesting that lincRNA-p21 is a target of p53 and is a member of the p53-dependent DNA damage response pathway in CLL and lymphoma [58].

### 8.3. LncRNA DLEU1 and LncRNA DLEU2

DLEU1 and DLEU2 (deleted in leukemia 1 and 2) lncRNA genes are located at the chromosome 13q14.3 region that is recurrently deleted in hematological malignancies, particularly in CLL. Deletion 13q14 is the most common cytogenetic aberration in CLL, present in more than 50% of patients and in around 30% of patients as a sole cytogenetic abnormality (del13q-only) [200]. It occurs more frequently in patients with mutated IGHV and low ZAP-70 expression [201,202]. It was originally observed that del13q14 is an independent prognostic marker of more indolent clinical course of CLL, since del13q-only cases experienced longer time to first treatment and overall survival [200,201]. However, this did not apply to all patients and further studies revealed that del13q14 is heterogeneous in size and that at least two subtypes of del13q14 exist: 1) small 13q14.3 deletions, encompassing a minimal deleted region which contains DLEU2 gene and the first exon of DLEU1 gene and 2) larger deletions involving 13q14.1-q14.2 band containing tumor suppressor RB1 locus [203]. The latter is associated with worse prognosis in the context of del13q-only CLL [204].

DLEU1 and DLEU2 are arranged in a head-to-head manner 300bp apart and are transcribed in opposite directions; the minimal deleted region of del13q14 encompasses the first exon of both genes [108]. DLEU2 is a host gene of miR-15a and miR-16-1, which are deleted or down-regulated in the majority of CLL patients [109,205]. DLEU2 acts as a tumor suppressor by negatively regulating the progression through the cell cycle. This function is mediated by miR-15a and miR-16-1 which down-regulate G1 phase cyclins E1 and D1, through binding to conserved miR-15a/miR-16-1 target sites in their 3′ UTRs [138]. In addition, DLEU2 is involved in regulation of apoptosis and this effect is also mediated by miR-15a and miR-16-1. It has been demonstrated that the levels of miR-15a and miR-16-1 are inversely correlated with the expression of BCL2 antiapoptotic protein in CLL cells and that both miRNAs repress BCL2 at post-transcriptional level through direct interaction with the 3′ UTR of BCL2 mRNA [109]. Thus, functional loss of miR-15a and miR-16-1 has pro-proliferative and anti-apoptotic effect and plays a crucial role in pathogenesis of CLL.

Besides DLEU1 and DLEU2, tumor-suppressor mechanisms at 13q14.3 involve several protein-coding tumor suppressor genes and pathogenesis of CLL probably results from concerted action of these elements [205,206]. Indeed, it has been demonstrated that DLEU1 and DLEU2 and their neighboring tumor-suppressor genes are epigenetically deregulated in the majority of CLL patients. A significantly lower DNA methylation at transcriptional start sites of DLEU1 and DLEU2 was observed in CLL cells in comparison to non-malignant B cells, as well as histone modifications associated with transcription. DNA demethylation level was positively correlated with the expression of DLEU1 and DLEU2, albeit negatively with the expression of neighboring tumor-suppressors, suggesting *in cis* co-regulation of these genes and their functional connection [205]. Indeed, both miR-15a/miR-16-1 and tumor-suppressor genes at 13q14.3 have been found to be involved in the modulation of NFκB signaling pathway, either as activators or repressors [205,206].

### 8.4. LncRNA BM74240

LncRNA BM74240 (GATA6-AS1) is located on chromosome18q11.2 in an antisense orientation to a neighboring GATA6 gene and is completely embedded in a CpG island. The analyses of methylation status of BM74240 promoter in CLL revealed that, while methylation was absent in normal B cells, BM74240 was completely or partially methylated in four out of seven CLL cell lines examined and in around 40% of primary CLL samples at diagnosis, implying that methylation is tumor-specific. Promoter methylation in CLL cell lines inversely correlated with BM74240 expression and a trend towards lower expression of neighboring GATA6 gene in BM74240-methylated cell lines was observed. In addition, BM74240 methylation in primary CLL samples was associated with higher lymphocyte count and older age at diagnosis, as well as with advanced Rai stage in the group with low-risk cytogenetics. Methylation of BM74240 was also associated with that of miR-129-2 at chromosome 11q11.2, which is frequently methylated in CLL at diagnosis, implying their cooperation in leukemogenesis [98]. Furthermore, stable overexpression of BM74240 in a completely methylated CLL cell line via lentiviral transduction resulted in reduction of proliferation and enhanced apoptosis through caspase-9-dependent intrinsic apoptotic pathway, indicating tumor suppressive function of BM74240 in CLL [98].

### 8.5. LncRNA TRERNA1

LncRNA TRERNA1 gene (translational regulatory lncRNA 1; also known as LINC00651, treRNA, ncRNA-a7) is located on chromosome 20q13.3. Its transcript contains two exons, is spliced and polyadenylated and has been reported to affect the expression of its targets, which may be cell type specific, at both transcriptional and translational level [43,207]. In CLL cells of asymptomatic, previously untreated patients, TRERNA1 was found to be overexpressed when compared to normal B cells and high expression level was associated with unfavorable prognostic markers (unmutated IGHV, high ZAP70 expression) and with shorter time to first treatment [144]. Among patients who received chemotherapy, high TRERNA1 expression was associated with shorter progression-free survival and overall survival in the group treated with fludarabine plus cyclophosphamide, but not in the group treated with fludarabine alone. In vitro studies on CLL cell line overexpressing exogenous TRERNA1 showed that TRERNA1 impairs the induction of DNA damage following exposure to chemotherapeutic agents, which may account for poor response to therapy in high-expressing patients [144].

### 8.6. LncRNA AC092652.2-202

LncRNA AC092652.2-202 is the main transcript of transcribed ultra-conserved region 70 (uc.70), located in an intronic region of ARHGAP15 gene (chr2q22.2-q22.3) in antisense orientation. High expression of both ARHGAP15 and AC092652.2-202 was found to be associated with shorter time to first treatment in CLL patients. AC092652.2-202 level prognostic value for TTFT was independent of IGHV mutational status and the disease stage in the whole cohort that was investigated, as well as in Binet A stage patients only. In addition, genes potentially modulated by AC092652.2-202 were enriched for pathways implicated in CLL pathogenesis, such as p53, apoptosis and NFκB [90].

### 8.7. Lnc-TOMM7-1, lnc-KIAA1755-4, lnc-IRF2-3, lncRNA ZNF667-AS1

A comprehensive study of lncRNA expression profile in Binet A stage CLL patients identified 24 lncRNAs deregulated in CLL compared to normal B cells. The most prominent down-regulation was found for lnc-TOMM7-1, which is mapped to chromosome 7p, antisense to interleukin-6 gene (Il-6), suggesting its involvement in Il-6 transcriptional regulation. Since Il-6 functions as an autocrine growth factor in CLL, this finding implies that lncTOMM7-1 plays a role in pathogenesis of the disease. In addition, in this study differential expression of lncRNAs in prognostic subgroups defined by IGHV mutational status, ZAP70 and CD38 expression, NOTCH1 mutations and cytogenetic aberrations was also determined and lncRNAs recurrently associated with adverse prognostic markers were identified, such as lnc-KIAA1755-4, lnc-IRF2-3 and lncRNA ZNF667-AS1. Analysis of correlation between lncRNA expression and time to first treatment pointed to lnc-KIAA1755-4 and lnc-IRF2-3 that, in combination, determined the best predictive model for TTFT. This model is independent from other prognostic markers and defines three risk groups (low, intermediate and high), where patients with low expression of both lncRNAs have the longest TTFT. Lnc-KIAA1755-4 is processed from the intron of small nucleolar host gene 17 (chromosome 20q11.23) and is located in the nucleolus where it regulates post-transcriptional modifications of rRNAs. The gene set enrichment analysis of lnc-KIAA1755-4 revealed enrichment of genes associated with translation, transcription, chromosome and telomere maintenance and telomeres packaging. On the other hand, for lnc-IRF2-3 (chromosome 4q35) enrichment of genes related to amino acid, sugar and lipid metabolism was shown [152].

Details on the role of lncRNAs deregulated in CLL are found in Table 1.

## 9. Conclusions

This review summarizes the latest research data on various lncRNAs whose expression is deregulated in leukemias. The overall conclusion that can be outlined from these studies is that lncRNAs have a great impact on the leukemic process. Additionally, based on the fact that aberrant expression of lncRNAs is cell and tissue specific, as well as specific for the exact differential stage, each lncRNA must be studied separately in each leukemia type. As can be seen from the presented data, the same lncRNA can have opposite functions, as oncogene or tumor-suppressor, depending on the particular type of leukemia.

Apart from the fact that lncRNAs play a major role in the pathogenesis of leukemia, some have already been recognized as potential molecular marker significant for diagnosis and prognosis of leukemias. As the classification of leukemia into risk groups and stages of leukemia progression is based primarily on specific genetic and molecular markers, monitoring of lncRNAs expression pattern can be an additional tool that can be used for the more precise stratification of patients. Moreover, since lncRNAs play an important role in crucial cellular processes, they can be used as targets for the design and application of specific therapy.

Thus, comprehensive genomics and transcriptomics profiling is the course that modern hematology should pursue in order to achieve the goal of a personalized therapeutic approach for each individual leukemic patient.

## Figures and Tables

**Table 1:** Long non-coding RNAs in leukemia.

Name of lncRNA	Expression in Leukemia Type	Function	Target	Effect	Prognostic Impact	References
AC092652.2-202	CLL-up-regulated	Oncogene			Increased expression is associated with adverse prognosis	[90]
ANRIL	AML-up-regulated	Oncogene	PRC1, PRC2	repression of INK4b-ARF-INK4a tumor-suppressor locus, increased proliferation	Increased expression is associated with adverse prognosis	[91,92]
			ADIPOR1	regulates glucose metabolism		[93]
	ALL-up-regulated	Oncogene	miR-7-5p	Accelerates proliferation and inhibits apoptosis	Increased expression is associated with adverse prognosis	[94]
BALR-2	ALL-up-regulated	Oncogene Drug resistance			Poor overall survival	[95]
BALR-6	ALL with MLL rearrangements-up-regulated	Oncogene	*SP1* and *CREB1*	Promotes proliferation and cell survival		[96]
BGL3	CML-down-regulated	Tumor suppressor	miR-17, miR-20a, miR-20b, miR-93, miR-106a, miR-106b	Alters the function of PTEN tumor suppressor		[97]
BM74240	CLL-down-regulated	Tumor suppressor			Increased expression is a marker of good prognosis	[98]
CCDC26	AML-up-regulated	Oncogene	c-KIT	Induces cell proliferation	Increased expression is associated with adverse prognosis	[99,100,101]
CRNDE	CLL-down-regulated	Tumor suppressor	miR-28	suppresses proliferation, induces apoptosis		[102]
	AML-up-regulated	Oncogene	miR-181 miR-136-5p	Promoting proliferation, blocking myeloid differentiation	Increased expression is associated with adverse prognosis	[103,104,105,106]
		Drug resistance	MRP1			[107]
DELU2	CLL-down-regulated	Tumor suppressor	miR-15a, miR-16-1	suppresses proliferation, induces apoptosis		[108,109]
GAS5	CLL-down-regulated	Tumor suppressor	miR-222	Suppresses proliferation		[88]
	AML-down-regulated	Tumor suppressor	miR-222		Low expression associated with inferior outcome in AML-NK	[85,110]
	ALL-up-regulated	Drug resistance	Glucocorticoid receptor	Increases glucocorticoid resistance	Higher risk of short-term relapse	[86,87]
H19	AML-up-regulated	Oncogene	ID2 miR-19a, miR-19b, miR-29a-3p	proliferation, apoptosis	Increased expression is associated with adverse prognosis	[111,112,113]
	CML-up-regulated	Oncogene Drug resistance	c-MYC		Increased expression is associated with disease progression and poor prognosis	[97,114]
	ALL-up-regulated	Oncogene	miR-326			[115]
HOTAIR	AML-up-regulated	Oncogene	p15^INK4b^ (CDKN2B) miR-193a and c-KIT	gene repression self-renewal	Increased expression is associated with adverse prognosis	[116,117,118]
	CML-up-regulated	Drug resistance	MRP1			[119]
	B-ALL-up-regulated					[120]
HOTAIRM1	AML-up-regulated	Oncogene	HOXA1, HOXA4, CD11b, CD18	chromatin modification, myeloid differentiation	Increased expression is associated with adverse prognosis	[121,122]
			miR-20a, miR-106b and miR-125b	autophagy		[123]
	APL-down-regulated	Tumor suppressor		regulates PML-RARa degradation	expression is up-regulated during ATRA-induced granulocytic differentiation promoting cell-cycle progression	[124]
HOXA-AS2	ALL-up-regulated	Drug resistance	HOXA3/EGFR/Ras/Raf/MEK/ERK pathway	Decreases glucocorticoid sensitivity		[125]
IRAIN	AML-down-regulated	Tumor suppressor	IGF1R promoter	Suppresses cell proliferation	down-regulated in patients with poor prognosis	[99,126]
LAMP-AS1	MLL rearrangement leukemias-up-regulated	Oncogene	LAMP5		Reduced 5-year leukemia-free survival	[95]
LEF1-AS1	CLL-up-regulated	Oncogene	LEF1	increases survival inhibits apoptosis		[127]
	AML-down-regulated	Tumor suppressor	P21^Cyp1^(CDKN1A) p27^Kip1^(CDKN1B)	Suppresses proliferation		[128]
LincRNA-p21	CLL-down-regulated	Tumor suppressor	p53 hnRNP-K	Induces apoptosis		[129,130]
LUNAR1	ALL-up-regulated	Oncogene	IGF1R	Increases proliferation	Poor prognosis in T-ALL	[131,132]
MALAT1	CLL-up-regulated	Oncogene	EZH2	Induces proliferation, suppresses apoptosis		[72]
	AML-up-regulated	Oncogene Drug resistance	miR-96	Increases proliferation		[73,75]
	CML-up-regulated	Oncogene	miR-328	Increases proliferation		[77]
	ALL-up-regulated	Oncogene	miR-205	Induces proliferation, suppresses apoptosis		[76]
MEG3	AML-down-regulated	Tumor suppressor	p53	Inhibits tumorigenesis via p53 dependent and independent way	Increased expression is a marker of good prognosis	[133,134,135]
		Drug resistance	miR-155			[136]
	CML-down-regulated	Drug resistance	miR-21			[137]
MIAT	CLL-up-regulated	Oncogene	OCT4	Increased cell growth, suppression of apoptosis	Increased expression is associated with adverse prognosis	[138]
NEAT1	CLL-down-regulated	Tumor suppressor	p53	Induces apoptosis		[58,59]
	AML-down-regulated	Tumor suppressor	miR-23a-3p miR-338-3p PTEN	suppresses proliferation, induces apoptosis	Overexpressed in APL where PML-RARa fusion protein inhibits NEAT1 expression	[61,62,63]
	CML-up-regulated	Oncogene	SFPQ-component of paraspeckles	suppresses apoptosis		[62]
PANDAR	AML-up-regulated	Oncogene	NF-YA transcription factor	Promotes cell survival	Increased expression is associated with adverse prognosis	[139,140]
PVT1	AML-up-regulated	Oncogene	c-MYC	Induces proliferation, suppresses apoptosis	Overexpression in APL and AML with t(8;21) indicates adverse prognosis	[141,142,143]
RP11-137H2.4	ALL-up-regulated	Drug resistance	NRAS/BRAF/NF-κB MAPK cascade members	Increases glucocorticoid resistance		[95]
TRERNA1	CLL-up-regulated	Oncogene			Increased expression is associated with adverse prognosis	[144]
TUG1	AML-up-regulated	Oncogene	AURKA miR-221-3p	increases cell proliferation, suppresses apoptosis	Increased expression is associated with adverse prognosis	[145,146]
		Drug resistance	miR-34a			[147]
UCA1	AML-up-regulated	Oncogene	p27^Kip1^ (CDKN1B)	Induces cell proliferation	Overexpressed in AML patients positive for CEPBA mutations	[148]
			miR-126	Increases viability, migration and invasion		[149]
		Drug resistance	miR-125			[150]
	CML-up-regulated	Drug resistance	miR-16			[151]
ZNF667-AS1	CLL-up-regulated	Oncogene			Increased expression is associated with adverse prognosis	[152]
	AML-up-regulated	Oncogene	miR-206	Increased proliferation and invasion of leukemic cells	Increased expression is associated with adverse prognosis	[153,154]
	APL-down-regulated	Tumor suppressor				[155]

## Data Availability

Not applicable.
